# The balance between N1 and N2 neutrophils implications for breast cancer immunotherapy: a narrative review

**DOI:** 10.1097/MS9.0000000000003361

**Published:** 2025-05-12

**Authors:** Emmanuel Ifeanyi Obeagu

**Affiliations:** Department of Biomedical and Laboratory Science, Africa University, Mutare, Zimbabwe

**Keywords:** breast cancer, immunotherapy, N1 neutrophils, N2 neutrophils, tumor microenvironment

## Abstract

Neutrophils, essential components of the innate immune system, exhibit remarkable plasticity in the tumor microenvironment, shifting between anti-tumoral (N1) and pro-tumoral (N2) phenotypes. This functional dichotomy is particularly significant in breast cancer, where N1 neutrophils contribute to tumor suppression by enhancing cytotoxicity and immune activation, while N2 neutrophils promote tumor progression through immunosuppression, angiogenesis, and metastasis. The tumor microenvironment, driven by factors such as TGF-β, IL-6, and hypoxia, orchestrates this polarization, profoundly influencing disease progression and therapeutic outcomes. The interplay between neutrophil polarization and breast cancer immunotherapy presents both challenges and opportunities. Pro-tumoral N2 neutrophils often hinder the efficacy of immune checkpoint inhibitors and other immunotherapies by suppressing T-cell function and facilitating tumor immune evasion. Conversely, strategies to reprogram neutrophils toward the N1 phenotype, including TGF-β inhibitors, CXCR2 antagonists, and epigenetic modulators, show promise in restoring anti-tumoral activity. Novel approaches, such as nanoparticle-mediated delivery of neutrophil-targeting agents, further expand the potential for precision immunotherapy by selectively modulating neutrophil phenotypes.

## Introduction

Breast cancer is one of the most prevalent malignancies worldwide, representing a significant public health challenge due to its heterogeneity and complex interactions with the immune system^[^1^]^. While advances in early detection and targeted therapies have improved outcomes, resistance to treatment and tumor progression remains substantial hurdles. Immunotherapy, particularly immune checkpoint inhibitors, has transformed cancer care, yet its efficacy in breast cancer has been limited. Recent research highlights the critical role of neutrophils, specifically their polarization into N1 and N2 phenotypes, in shaping the tumor microenvironment and influencing therapeutic outcomes^[^2,3^].^ Neutrophils, traditionally viewed as terminally differentiated first responders in the immune system, are now recognized for their functional plasticity and diverse roles in cancer biology^[^4^]^. These granulocytes are recruited to tumor sites by various chemokines, where they interact dynamically with other immune cells, tumor cells, and stromal components. Their phenotype and function are dictated by the tumor microenvironment, with N1 neutrophils exerting anti-tumoral effects, such as cytotoxicity and immune activation, and N2 neutrophils facilitating tumor growth through immunosuppression and angiogenesis. This duality underscores the significance of the N1/N2 balance in cancer progression^[^5^].^ In breast cancer, the tumor microenvironment is rich in factors like transforming growth factor-beta (TGF-β), interleukin-6 (IL-6), and hypoxia-inducible factor-1 alpha (HIF-1α), which skew neutrophils toward the N2 phenotype. This pro-tumoral polarization supports various processes, including immune evasion, extracellular matrix remodeling, and pre-metastatic niche formation, ultimately leading to enhanced tumor invasiveness and metastasis. Conversely, strategies to sustain or induce the N1 phenotype hold promise for disrupting these tumor-promoting pathways and restoring effective anti-tumor immunity.^[^6-8^]^

**HIGHLIGHTS**

- Neutrophils in breast cancer exhibit distinct N1 (anti-tumoral) and N2 (pro-tumoral) phenotypes, influencing tumor progression and response to therapy.
- N2 neutrophils contribute to immune suppression and metastasis.
- Reprogramming N2 neutrophils to N1 could enhance immunotherapy efficacy.
- Neutrophil-targeted therapies may complement existing breast cancer treatments.
- Advancing neutrophil polarization research could lead to personalized, more effective cancer therapies.

The neutrophil-to-lymphocyte ratio (NLR), a readily available clinical marker, has gained attention as a prognostic indicator in breast cancer. Elevated NLR often reflects an abundance of pro-tumoral neutrophils and a poor prognosis. This association underscores the clinical relevance of targeting neutrophil subsets to improve outcomes. The therapeutic potential of neutrophil modulation extends beyond prognostication, offering avenues to enhance the efficacy of existing treatments and address resistance mechanisms^[^9^].^ Despite the growing interest in neutrophils as therapeutic targets, their high plasticity poses significant challenges. N1 and N2 phenotypes are not static; they exist on a spectrum and can transition based on environmental cues. Current research focuses on elucidating the signaling pathways and transcriptional networks that govern N1 and N2 polarization, as well as the interactions between neutrophils and other immune or tumor cells^[^10,11^].^ Checkpoint inhibitors, such as anti-PD-1 and anti-CTLA-4 therapies, have shown limited success in breast cancer, partly due to the immunosuppressive actions of N2 neutrophils. These cells express PD-L1 and other molecules that dampen T-cell responses, creating a barrier to effective immunotherapy. Targeting N2 neutrophils or reprogramming them into N1 phenotypes represents a promising strategy to enhance the therapeutic efficacy of these agents. Additionally, combining checkpoint inhibitors with neutrophil-modulating agents may synergistically restore anti-tumoral immunity^[^12^]^.

Innovative approaches, such as nanoparticle-mediated drug delivery, are being explored to selectively target neutrophils and influence their phenotype. Nanoparticles can deliver drugs directly to neutrophils or the tumor microenvironment, minimizing off-target effects and maximizing therapeutic impact. These technologies also enable the delivery of combination therapies, offering a multifaceted approach to overcoming the challenges posed by neutrophil plasticity^[^13,14^]^. Another promising avenue lies in the use of small-molecule inhibitors and epigenetic modulators to shift neutrophils toward an N1 phenotype. These agents target specific signaling pathways or transcription factors involved in neutrophil polarization, providing precision in modulating their function. For instance, TGF-β inhibitors block one of the primary signals driving N2 polarization, while epigenetic drugs can reprogram gene expression patterns to favor anti-tumoral activity^[^15^]^.

## Aim

The aim of this review is to explore the critical role of N1 and N2 neutrophils in breast cancer progression and therapy.

## Rationale

The tumor microenvironment is highly complex and dynamic, with immune cells playing a pivotal role in either supporting or inhibiting cancer progression. Among these immune cells, neutrophils are particularly significant due to their ability to shift between two distinct phenotypes: N1 and N2. The N1 phenotype is characterized by anti-tumoral properties, while the N2 phenotype supports tumor progression, metastasis, and immune evasion. Understanding the balance between these phenotypes is crucial for improving breast cancer therapy. Recent studies have highlighted that neutrophils, traditionally considered as innate immune responders, contribute more substantially to tumor development than previously understood. N2 neutrophils, in particular, foster an immunosuppressive environment by secreting cytokines that impair the anti-tumoral immune response^[^16,17^]^. Conversely, N1 neutrophils are involved in direct tumor cell killing and activation of other immune cells. The dynamic shift between these two phenotypes within the tumor microenvironment presents both challenges and opportunities for therapeutic intervention. Targeting neutrophil polarization could represent a novel and strategic approach to breast cancer treatment, especially in overcoming resistance to current therapies such as immune checkpoint inhibitors. By reprogramming N2 neutrophils to the N1 phenotype or modulating their functions, it may be possible to enhance the effectiveness of existing treatments and reduce cancer progression. Thus, a deeper understanding of neutrophil polarization and its implications for breast cancer immunotherapy is not only timely but critical for advancing therapeutic strategies that are both more precise and personalized^[^18,19^]^.

## Review methodology

### Literature search

A comprehensive search was conducted across multiple academic databases, including PubMed, Scopus, Google Scholar, and Web of Science. The search terms used included “N1 and N2 neutrophils,” “breast cancer,” “tumor microenvironment,” “neutrophil polarization,” “immunotherapy in breast cancer,” and “neutrophil dynamics in cancer.” The search was restricted to studies published in peer-reviewed journals in the past 10 years (2013–2023) to ensure relevance and currency of the data.

### Inclusion and exclusion criteria

The studies selected for inclusion in this review met the following criteria:

- Investigated the role of neutrophils in breast cancer progression and immunotherapy.
- Focused on the N1 and N2 neutrophil phenotypes and their polarization mechanisms.
- Provided data on the tumor microenvironment and immune responses related to neutrophils in breast cancer.
- Included *in vitro, in vivo*, and clinical studies.

Exclusion criteria included the following:

- Studies focusing on neutrophils in other cancers or unrelated to breast cancer.
- Articles not available in English or lacking full-text access.
- Research articles older than 10 years (unless they provided foundational insights).

### Quality assessment

Each article was assessed for quality based on the strength of its methodology, including the study design, sample size, and data analysis methods. The quality of evidence was graded according to a standard evidence hierarchy, with clinical trial data given higher weight than observational studies or preclinical models. Articles were also evaluated for the clarity of their findings and the reproducibility of their results.

### Neutrophil polarization: N1 vs. N2 phenotypes

Neutrophils, long regarded as mere responders to infection and inflammation, have emerged as complex players in cancer biology, particularly in their ability to polarize into distinct functional states known as N1 and N2 phenotypes. This polarization reflects their remarkable plasticity and is heavily influenced by the tumor microenvironment. The N1 phenotype is characterized by its anti-tumoral properties. These neutrophils are potent immune activators, capable of producing high levels of reactive oxygen species (ROS) and nitric oxide (NO), both of which contribute to direct tumor cell killing. Furthermore, N1 neutrophils amplify immune responses by releasing pro-inflammatory cytokines such as tumor necrosis factor-alpha (TNF-α) and interferon-gamma (IFN-γ), thereby recruiting and activating other immune cells to mount a robust defense against tumor growth^[^18^].^ In contrast, N2 neutrophils are pro-tumoral, actively supporting cancer progression through various mechanisms. They secrete vascular endothelial growth factor (VEGF) and matrix metalloproteinases (MMPs), which promote angiogenesis and facilitate tumor invasion and metastasis. Additionally, N2 neutrophils contribute to immune suppression by expressing checkpoint molecules like PD-L1, which inhibit T-cell activity, and by releasing anti-inflammatory cytokines, including interleukin-10 (IL-10). This phenotype not only fosters a permissive environment for tumor survival but also hinders the efficacy of immunotherapeutic interventions^[^19,20^].^ The dynamic shift between N1 and N2 phenotypes is orchestrated by the tumor microenvironment, with key factors such as transforming growth factor-beta (TGF-β), interleukin-6 (IL-6), and hypoxia-inducible factor-1 alpha (HIF-1α) playing pivotal roles. For example, TGF-β signaling strongly skews neutrophils toward the N2 phenotype, suppressing their cytotoxic functions while enhancing their pro-tumoral activities. This ability to transition between N1 and N2 states underscores the complexity of neutrophils in cancer and highlights their dual role as both defenders and facilitators of disease. Understanding the molecular cues that govern this polarization is essential for developing strategies to modulate neutrophil behavior, shifting the balance toward anti-tumoral activity and improving therapeutic outcomes in breast cancer and beyond^[^21,22^]^.

### Neutrophil dynamics in breast cancer

Neutrophils are dynamic participants in the progression of breast cancer, profoundly influenced by the tumor microenvironment. They are recruited to the tumor site by various chemokines, including CXCL1, CXCL2, and CXCL8, which are abundantly secreted by tumor and stromal cells. Upon arrival, these neutrophils do not act uniformly; instead, their function and phenotype are shaped by a multitude of factors, including the tumor’s molecular subtype, its stage, and the surrounding stromal and immune cell interactions^[^23^].^ In early-stage breast cancer, neutrophils may exhibit a predominantly anti-tumoral N1 phenotype. These neutrophils contribute to immune surveillance by producing reactive oxygen species (ROS) and nitric oxide (NO), inducing direct cytotoxicity against tumor cells. Additionally, they release pro-inflammatory cytokines that enhance the recruitment of effector immune cells like CD8 + T cells, fostering a more effective anti-tumor immune response. However, as the tumor progresses, the microenvironment begins to suppress N1 neutrophils while promoting the differentiation and accumulation of pro-tumoral N2 neutrophils^[^24^].^ The role of N2 neutrophils becomes increasingly prominent in advanced breast cancer. These neutrophils enhance tumor progression by promoting angiogenesis through vascular endothelial growth factor (VEGF) secretion and facilitating extracellular matrix remodeling via matrix metalloproteinases (MMPs). Furthermore, N2 neutrophils contribute to immune evasion by expressing high levels of PD-L1 and releasing immunosuppressive cytokines like IL-10, which inhibit T-cell activity and sustain a tolerogenic microenvironment. This shift toward an immunosuppressive and pro-metastatic phenotype not only supports primary tumor growth but also aids in the establishment of metastatic niches, particularly in organs like the lungs and bones, common sites for breast cancer metastases^[^25^]^.

Hypoxia within the tumor microenvironment plays a significant role in regulating neutrophil dynamics. Hypoxia-inducible factors (HIFs) drive the polarization of neutrophils toward the N2 phenotype by upregulating genes involved in angiogenesis, migration, and immune suppression. Similarly, elevated levels of TGF-β within the tumor microenvironment further amplify the pro-tumoral activities of neutrophils, creating a self-sustaining cycle of tumor progression^[^26^].^ Clinical evidence further highlights the significance of neutrophil dynamics in breast cancer. A high neutrophil-to-lymphocyte ratio (NLR) in peripheral blood has been consistently associated with a poor prognosis in breast cancer patients. This observation underscores the role of neutrophils as not only biomarkers of disease progression but also as active contributors to the tumor’s pathophysiology^[^25^]^. Targeting the mechanisms that skew neutrophils toward the N2 phenotype has emerged as a promising therapeutic strategy. Approaches such as TGF-β inhibition, CXCR2 antagonism, and epigenetic modulation aim to prevent pro-tumoral polarization or reprogram N2 neutrophils back to the anti-tumoral N1 state. Furthermore, combining these strategies with conventional therapies or immunotherapies could significantly enhance their efficacy, particularly in resistant or advanced-stage breast cancer^[^27^].^ The dual nature of neutrophil dynamics in breast cancer underscores the importance of context in their functional roles. While they can act as potent defenders of the host in their N1 state, their transition to the N2 phenotype transforms them into facilitators of disease progression. This plasticity represents both a challenge and an opportunity in the realm of breast cancer research, offering potential pathways for innovative therapeutic interventions^[^27^]^.

### The balance between N1 and N2 neutrophils in breast cancer

The balance between N1 and N2 neutrophils represents a critical axis in the regulation of breast cancer progression and the response to therapy. This balance is not static but dynamic, shaped by the interactions between neutrophils and the tumor microenvironment. The polarization of neutrophils toward either the N1 or N2 phenotype can significantly influence tumor growth, metastasis, and the efficacy of therapeutic interventions^[^28^].^ N1 neutrophils are anti-tumoral by nature. They produce high levels of reactive oxygen species (ROS), nitric oxide (NO), and pro-inflammatory cytokines, which contribute to the direct killing of tumor cells and the activation of other immune cells. These neutrophils also enhance the recruitment of cytotoxic T cells and other effector immune cells, thereby amplifying anti-tumoral immune responses. In the context of breast cancer, a robust presence of N1 neutrophils is associated with better prognoses and heightened responsiveness to immune-based therapies^[^29^].^ Conversely, N2 neutrophils support tumor progression and metastasis. These cells promote angiogenesis through the secretion of vascular endothelial growth factor (VEGF) and facilitate extracellular matrix remodeling via matrix metalloproteinases (MMPs). Furthermore, N2 neutrophils contribute to immune suppression by expressing checkpoint molecules like PD-L1 and releasing immunosuppressive cytokines such as interleukin-10 (IL-10) and transforming growth factor-beta (TGF-β). This pro-tumoral phenotype not only fosters a permissive microenvironment for tumor growth but also undermines the effectiveness of immunotherapies, including immune checkpoint inhibitors^[^30^]^.

The tumor microenvironment plays a decisive role in tipping the balance between N1 and N2 phenotypes. Factors such as TGF-β, hypoxia-inducible factor-1 alpha (HIF-1α), and interleukin-6 (IL-6) are instrumental in driving N2 polarization. Hypoxic conditions within the tumor core, for example, skew neutrophils toward the N2 phenotype by upregulating pro-angiogenic and immunosuppressive genes. This dynamic creates a feedback loop where N2 neutrophils further modify the microenvironment to sustain their pro-tumoral activities^[^31^].^ Therapeutically targeting the N1/N2 balance is emerging as a promising strategy in breast cancer treatment. By inhibiting key drivers of N2 polarization, such as TGF-β signaling or CXCR2-mediated recruitment, it is possible to reduce the population of pro-tumoral neutrophils. Conversely, strategies that promote the N1 phenotype, such as the use of cytokines like IFN-γ or agents that enhance ROS production, can reprogram neutrophils to support anti-tumoral immunity^[^32^].^ The clinical implications of this balance are profound. A high neutrophil-to-lymphocyte ratio (NLR) in peripheral blood, often indicative of an N2-dominated response, correlates with poor outcomes in breast cancer patients. This observation underscores the need for diagnostic tools that assess neutrophil polarization within the tumor microenvironment, enabling more precise and personalized therapeutic approaches^[^33^].^ The ability to modulate the N1/N2 balance could also enhance the efficacy of combination therapies. For instance, reprogramming neutrophils toward the N1 phenotype could synergize with immune checkpoint inhibitors or cancer vaccines, improving their effectiveness even in immunosuppressive tumor settings. Additionally, novel drug delivery systems, such as nanoparticles, offer the potential to selectively target neutrophils within the tumor microenvironment, minimizing off-target effects and maximizing therapeutic benefits^[^34^]^.

### Molecular mechanisms and research progress in balancing N1 and N2 neutrophils in breast cancer

In the evolving landscape of breast cancer immunology, the once-overlooked neutrophil has become a focal point of intense investigation. Far from being passive bystanders, neutrophils now occupy a central role in shaping tumor fate. Their dichotomous polarization into either N1 (anti-tumorigenic) or N2 (pro-tumorigenic) subsets reflects the complex interplay between innate immunity and the tumor microenvironment (TME). The crux of modern immunotherapeutic innovation lies in understanding the molecular underpinnings of this polarization – and, more importantly, how to tilt the balance in favor of N1 neutrophils to harness their tumor-fighting potential^[^24^].^ At the molecular level, the polarization of neutrophils is orchestrated by a rich tapestry of signaling molecules and transcriptional regulators. Transforming growth factor-beta (TGF-β) stands out as a key driver of N2 phenotype induction. Its immunosuppressive influence extends beyond neutrophils, but in their case, TGF-β acts as a powerful reprogramming agent, downregulating cytotoxic capabilities while promoting tissue remodeling, angiogenesis, and immunoevasion. By contrast, type I interferons, particularly interferon-beta (IFN-β), stimulate the polarization toward the N1 subtype. These interferons activate key transcription factors such as IRF9 and STAT1, leading to enhanced expression of pro-inflammatory cytokines, chemokines (like CXCL10), and increased ROS production – hallmarks of the N1 neutrophil^[^35^].^ Recent preclinical research has uncovered multiple checkpoints in this polarization process that are amenable to therapeutic manipulation. One of the most promising avenues involves targeting the TGF-β signaling axis. In murine models of breast cancer, pharmacologic blockade of TGF-β receptors or the use of neutralizing antibodies has shown success in reprogramming TANs (tumor-associated neutrophils) from an N2-like to an N1-like phenotype. These interventions not only suppressed tumor growth but also synergized with immune checkpoint blockade, reviving exhausted cytotoxic T cells within the TME^[^36^]^.

Simultaneously, researchers have explored exogenous stimulation with IFN-β as a way to push neutrophils toward an N1 identity. The administration of recombinant IFN-β, either systemically or through intratumoral delivery, has been shown to invigorate neutrophil anti-tumor functions while also acting on other immune subsets. This strategy offers the advantage of broadly activating the innate immune landscape but must be carefully calibrated to avoid systemic toxicity^[^37^].^ Another molecular pathway of interest involves the CXCR2 signaling cascade. N2 neutrophils often express high levels of CXCR2, which mediates their recruitment to the tumor site in response to chemokines like CXCL1 and CXCL8. Inhibition of CXCR2 signaling has been shown to reduce the infiltration of N2-like TANs while preserving or enhancing the recruitment of N1 counterparts. Small molecule CXCR2 antagonists are already under investigation in clinical trials for other malignancies and represent a translatable strategy for breast cancer as well^[^24^].^ Further, epigenetic modifiers are emerging as critical regulators of neutrophil plasticity. Inhibitors of histone deacetylases (HDACs) have demonstrated the ability to skew neutrophils toward an N1-like phenotype, potentially through modulation of gene expression programs related to immune activation. HDAC inhibition, when combined with immunotherapy, may offer a two-pronged attack – reprogramming both innate and adaptive immune compartments^[^35^].^ An exciting frontier in this field is the use of nanoparticle-based delivery systems to achieve localized neutrophil reprogramming. These technologies allow for the co-delivery of polarizing agents, such as IFN-β or TGF-β inhibitors, directly into the tumor site, minimizing systemic effects and enhancing therapeutic precision. Nanocarriers have also been employed to deliver siRNAs or miRNAs that target specific regulators of neutrophil function, opening up a realm of gene-silencing strategies to modulate neutrophil behavior^[^36^].^ Meanwhile, translational research has turned to biomarker identification as a means of stratifying patients based on TAN profiles. The neutrophil-to-lymphocyte ratio (NLR), while crude, remains a clinically relevant prognostic indicator. However, more sophisticated techniques – such as single-cell RNA sequencing, multiplex immunohistochemistry, and spatial transcriptomics – are revealing the true heterogeneity of TAN populations in breast cancer. These tools will be instrumental in designing personalized immunotherapeutic strategies based on neutrophil phenotypes^[^37^]^.

### Controlling the polarization and dynamics of N1 and N2 neutrophils in human breast cancer

In the realm of human breast cancer, the complexity of tumor-immune interactions is underscored by the versatile behavior of neutrophils. These short-lived yet highly adaptable immune cells are far from uniform in function; rather, they are sculpted by the tumor microenvironment into distinct phenotypes – namely, the anti-tumorigenic N1 and the pro-tumorigenic N2 neutrophils. The ability to control their polarization and dynamics holds promise for redefining immunotherapy outcomes in breast cancer. However, this control requires a deep understanding of both the molecular language they speak and the cues they respond to within the human host^[^2^].^ At the heart of neutrophil polarization lies the influence of tumor-derived cytokines, chemokines, and extracellular matrix components. In the human breast cancer microenvironment, TGF-β emerges as a dominant polarizing force toward the N2 phenotype. It dampens neutrophilic cytotoxicity and encourages the secretion of matrix metalloproteinases (MMPs), VEGF, and arginase – all facilitators of tumor progression and immune suppression. Thus, pharmacological inhibition of TGF-β signaling stands out as a viable strategy to interrupt this skewing. Several TGF-β inhibitors, such as galunisertib, are currently under investigation in clinical trials, and their integration with immunotherapy protocols could pivot neutrophil dynamics toward tumoricidal behavior^[^38^].^ In contrast, type I interferons, notably IFN-α and IFN-β, represent potent polarizers toward the N1 phenotype. These cytokines enhance the neutrophil’s arsenal of reactive oxygen species (ROS), TNF-α, and interleukin-12 (IL-12), fostering anti-tumor cytotoxicity and dendritic cell recruitment. Clinical manipulation of these pathways, such as through pegylated IFN-α or recombinant IFN-β therapy, may reinvigorate innate immune responses. Yet, in human subjects, dosing, toxicity, and delivery mechanisms remain challenges that demand precision, particularly in balancing efficacy with off-target inflammation^[^39^]^.

Beyond direct cytokine modulation, targeting chemokine-receptor axes offers another layer of control over neutrophil infiltration and fate. In breast cancer patients, N2-like neutrophils often express high levels of CXCR2, guiding their chemotactic migration to tumor sites rich in CXCL1, CXCL5, and CXCL8. CXCR2 inhibitors – already in development for inflammatory diseases – may thus serve dual roles in both reducing tumor infiltration by N2 neutrophils and limiting metastatic spread. In human models, such inhibitors are being paired with checkpoint inhibitors to enhance antitumor immune infiltration^[^40^].^ An additional strategy involves epigenetic reprogramming of neutrophils. Human neutrophils respond to modifications in chromatin accessibility through exposure to histone deacetylase (HDAC) inhibitors and DNA methylation modulators. For example, low-dose HDAC inhibitors like entinostat have shown potential in reactivating immune responses and may also shift neutrophil behavior toward an N1-like profile. The modulation of neutrophil transcriptional landscapes offers a promising, albeit complex, means of resetting immune tone within the tumor^[^41^].^ Metabolic reprogramming is another emergent concept gaining traction. Tumor-associated neutrophils (TANs) in breast cancer are metabolically active and can be influenced by the local availability of oxygen, glucose, and lipids. Studies in humanized models have shown that N1 neutrophils favor glycolytic metabolism, whereas N2 neutrophils lean toward oxidative phosphorylation. Therefore, targeting metabolic pathways – such as through inhibitors of fatty acid oxidation or glucose metabolism modulators – could potentially influence polarization states *in situ*^[^38^]^.

Importantly, a new wave of biomaterials and delivery technologies are being developed to localize and control the release of neutrophil-modulating agents directly within the tumor. For example, nanoparticles carrying TGF-β inhibitors or IFN-inducers have been designed to home in on breast tumors, releasing their payload in a controlled fashion, thereby influencing neutrophil polarization with minimal systemic exposure^[^39^].^ Furthermore, spatial and temporal mapping of neutrophils in human breast tumors using techniques like single-cell RNA sequencing and multiplex imaging has enabled the identification of neutrophil subclusters and their transitional states. This has shed light on how neutrophil states shift not just with molecular cues but also in response to therapy, such as chemotherapy or immunotherapy. For instance, patients receiving neoadjuvant chemotherapy may exhibit a transient spike in N1-like TANs, which can be leveraged through strategic timing of immunotherapy^[^40^].^ Lastly, personalized medicine approaches are gradually integrating neutrophil-based markers into prognostic and therapeutic decision-making. The neutrophil-to-lymphocyte ratio (NLR), while crude, has already demonstrated predictive value for immunotherapy outcomes. More refined markers, such as surface expression of CD177, CD274 (PD-L1), or LOX-1 on circulating neutrophils, are being explored to stratify patients based on neutrophil phenotype and guide therapy selection^[^41^]^.

### Latest clinical progress and future prospects in targeting neutrophils for breast cancer prevention and treatment

The clinical landscape of breast cancer treatment is rapidly evolving beyond traditional cytotoxic therapies into the nuanced realm of immune modulation. Among the various immune cells being explored, neutrophils have emerged as pivotal yet underappreciated players in both cancer progression and immune resistance. Once thought to be mere responders to infection and inflammation, neutrophils are now recognized as highly plastic cells capable of both suppressing and supporting tumor growth depending on their polarization. Recent strides in understanding neutrophil biology have spurred a new wave of clinical research focused on harnessing or reprogramming these cells for therapeutic gain in human breast cancer.

### Clinical translation

Translating insights about neutrophil polarization (N1 vs. N2) into clinical interventions has taken multiple promising directions. One of the most significant breakthroughs has come in TGF-β inhibition, which has been shown to shift tumor-associated neutrophils (TANs) from a pro-tumorigenic N2 state to a more aggressive, anti-tumor N1 phenotype. Clinical trials using TGF-β inhibitors, such as galunisertib and vactosertib, have demonstrated encouraging results in solid tumors, including breast cancer. While not yet approved, these agents have shown synergy with immune checkpoint inhibitors like anti-PD-1 antibodies, suggesting that dual therapy involving neutrophil reprogramming and adaptive immune activation could become a cornerstone of next-generation breast cancer therapy. Another noteworthy development involves CXCR2 antagonists, which target the chemokine axis that drives N2 neutrophil recruitment into the tumor microenvironment. In clinical studies, blocking CXCR2 has not only reduced the infiltration of immunosuppressive TANs but also improved the efficacy of checkpoint blockade by allowing T cells to thrive in a more permissive immune landscape. A phase Ib trial combining navarixin (a CXCR1/2 inhibitor) with pembrolizumab has reported favorable immune modulation in triple-negative breast cancer (TNBC), pointing to its translational potential. The application of interferon therapy, particularly IFN-β, has also progressed into early clinical phases. Recombinant IFN-β has been used in neoadjuvant settings to boost the N1 neutrophil response, increase dendritic cell cross-presentation, and promote T cell infiltration. However, its systemic toxicity profile remains a challenge, prompting ongoing research into tumor-targeted delivery systems using nanoparticles or liposomes to localize its activity and limit adverse effects. Additionally, biomarker-driven approaches are beginning to take hold in the clinical transformation of neutrophil-targeted strategies. For instance, neutrophil surface markers such as LOX-1 and CD274 (PD-L1) are under investigation as predictors of patient prognosis and therapeutic responsiveness. Integrating these markers into clinical diagnostics could allow for patient stratification, enabling the selection of individuals who are more likely to benefit from neutrophil-targeted therapies.^[^26,42-44^]^

### Prospect

As single-cell sequencing and spatial transcriptomics continue to unravel the heterogeneity of TANs in human breast tumors, researchers are uncovering multiple neutrophil subpopulations that transcend the binary N1/N2 model. This nuanced understanding will likely inform precision-based interventions that go beyond global neutrophil depletion and instead aim to modulate specific functional subsets. Emerging technologies such as engineered exosomes, microRNA delivery, and CRISPR-based gene editing offer exciting possibilities for fine-tuning neutrophil activity at the molecular level. For example, miR-223 and miR-155 – known regulators of neutrophil phenotype – are being explored for their potential to reprogram TANs *in situ*. Similarly, the manipulation of metabolic pathways, such as glycolysis and oxidative phosphorylation, may help push neutrophils toward an anti-tumor state while preserving their ability to combat infections. Moreover, the potential preventive applications of neutrophil-targeted therapies are beginning to surface. In high-risk individuals with BRCA mutations or chronic inflammatory states, preclinical models suggest that early modulation of neutrophilic inflammation may reduce tumor initiation and enhance immunosurveillance. Such preventive immunomodulation could represent a transformative strategy in oncology, shifting the goal from treatment to risk reduction. Importantly, the integration of neutrophil-targeted therapies with existing treatment regimens – chemotherapy, radiotherapy, and immunotherapy – will likely be key to their success. For instance, chemotherapy-induced neutropenia, once viewed solely as a side effect, is now being investigated as a therapeutic window to reshape the immune microenvironment before subsequent immunotherapy. Harnessing this opportunity could redefine the treatment sequence for optimal clinical impact.^[^24,45-47^]^

### Novel findings and insights in targeting neutrophils for breast cancer therapy

Recent years have seen a surge in discoveries that are reshaping how we view neutrophils within the tumor microenvironment, particularly in breast cancer. Traditionally overshadowed by lymphocytes and macrophages in cancer immunology, neutrophils are now recognized as highly dynamic cells capable of influencing tumor progression, immune evasion, and therapy resistance. Novel insights have expanded our understanding beyond the binary N1 (anti-tumor) and N2 (pro-tumor) classification, revealing a spectrum of phenotypes and functions that are tightly governed by spatial, temporal, and molecular contexts within the breast tumor ecosystem.

**1. The Discovery of Neutrophil Subpopulations With Unique Transcriptional Signatures**

One of the most groundbreaking findings in recent studies is the identification of distinct neutrophil subpopulations within breast tumors using single-cell RNA sequencing. These studies have shown that breast cancer-infiltrating neutrophils are not homogenous but instead exhibit transcriptional programs associated with antigen presentation, interferon signaling, reactive oxygen species generation, or immunosuppression. Some tumor-infiltrating neutrophils express high levels of PD-L1, suggesting a role in suppressing T-cell activity directly, while others display features of immature neutrophils with proliferative and stem-like properties, potentially contributing to tumor plasticity and therapy resistance^[^48^]^.

**2. NETosis as a Therapeutic Target**

Another novel insight is the pathological role of neutrophil extracellular traps (NETs) in breast cancer progression. NETs, composed of DNA and granule proteins, were once thought to be beneficial in trapping pathogens, but they are now known to facilitate metastasis by capturing circulating tumor cells (CTCs) and promoting their adhesion to distant organs. Inhibitors of PAD4, an enzyme critical for NET formation, have shown promise in preclinical models by reducing metastatic spread and enhancing the efficacy of checkpoint inhibitors. This has led to early-phase clinical investigations of PAD4 inhibitors as adjuvants in high-risk breast cancer^[^49^]^.

**3. Neutrophils as Predictive Biomarkers for Immunotherapy Response**

Recent clinical data suggest that neutrophil phenotypes can predict response to immunotherapy. High levels of circulating low-density neutrophils (LDNs), often associated with an N2-like profile, have been correlated with poor response to anti-PD-1/PD-L1 therapy in triple-negative breast cancer (TNBC). Conversely, a high neutrophil-to-lymphocyte ratio (NLR), traditionally considered a poor prognostic marker, is now being re-evaluated as a dynamic biomarker that could reflect shifts in neutrophil polarization during therapy. These findings underscore the need for real-time immune profiling to guide treatment decisions^[^50^]^.

**4. Targeting Metabolic Pathways to Reprogram Neutrophils**

A novel strategy that is gaining traction involves modulating the metabolic state of neutrophils. Emerging research shows that N2 neutrophils rely heavily on fatty acid oxidation and oxidative phosphorylation, while N1-like neutrophils prefer glycolysis. Agents that skew neutrophil metabolism – such as metformin or dichloroacetate (DCA) – have shown potential in reprogramming pro-tumor TANs into cytotoxic effector cells. These agents are particularly attractive because of their availability, safety profile, and potential for repurposing in clinical settings^[^51^]^.

**5. Engineering Neutrophils as Cellular Therapeutics**

Perhaps the most innovative frontier is the use of engineered neutrophils as delivery systems for anti-cancer agents. Leveraging the natural ability of neutrophils to home to inflammatory sites, researchers have designed nanoparticle-loaded neutrophils that carry chemotherapeutic drugs or immune modulators directly to tumor tissues. Some experimental systems have utilized neutrophil membranes to cloak nanoparticles, enhancing their biocompatibility and tumor-targeting efficiency. These biomimetic delivery systems offer a glimpse into the next generation of neutrophil-based cancer therapies, particularly for overcoming immune-excluded tumors^[^52^]^.

### Implications for immunotherapy

The dual nature of neutrophils, characterized by their polarization into N1 (anti-tumoral) and N2 (pro-tumoral) phenotypes, has profound implications for the efficacy of immunotherapy in breast cancer. Immunotherapies, particularly immune checkpoint inhibitors (ICIs) targeting PD-1, PD-L1, and CTLA-4, have revolutionized cancer treatment. However, their success in breast cancer has been limited, partly due to the immunosuppressive microenvironment fostered by N2 neutrophils^[^53^].^ N2 neutrophils impede immunotherapy by expressing high levels of PD-L1 and releasing cytokines like IL-10 and TGF-β, which suppress T-cell activation and proliferation. This creates a significant barrier to the efficacy of ICIs, as the restored T-cell activity central to these therapies is hindered by the immunosuppressive activities of N2 neutrophils. Additionally, N2 neutrophils contribute to immune evasion by promoting tumor-associated macrophages (TAMs) and regulatory T-cells (Tregs), further dampening anti-tumoral immune responses^[^20^].^ Strategies to modulate neutrophil phenotypes are emerging as a means to enhance immunotherapy. One promising approach involves targeting TGF-β, a critical driver of N2 polarization. TGF-β inhibitors can reduce the immunosuppressive functions of N2 neutrophils while fostering a microenvironment conducive to T-cell activation. Similarly, CXCR2 antagonists, which block the recruitment of neutrophils to the tumor site, can diminish the population of pro-tumoral neutrophils, thereby reducing immune suppression^[^54^].^ Combining neutrophil-modulating agents with ICIs is a particularly attractive strategy. Preclinical studies suggest that inhibiting the pro-tumoral functions of neutrophils can restore T-cell responses, thereby synergizing with checkpoint blockade therapies. For example, reducing neutrophil recruitment through CXCR2 inhibition has been shown to enhance the infiltration of cytotoxic T lymphocytes into tumors, amplifying the effects of ICIs.

Epigenetic modulation represents another innovative avenue for reprogramming neutrophils. Drugs targeting histone deacetylases (HDACs) or DNA methyltransferases (DNMTs) can alter gene expression patterns in neutrophils, shifting their phenotype from N2 to N1. These reprogrammed neutrophils can then support immunotherapy by enhancing anti-tumoral immunity through cytokine production and immune cell recruitment^[^55^].^ Nanoparticle-based drug delivery systems further expand the therapeutic potential by selectively targeting neutrophils or the tumor microenvironment. Nanoparticles can deliver TGF-β inhibitors, CXCR2 antagonists, or other modulators directly to the tumor site, minimizing systemic toxicity while maximizing therapeutic efficacy. These technologies also enable precise combination therapies, integrating neutrophil modulation with ICIs in a single treatment platform^[^56^].^ The implications of targeting neutrophils extend beyond checkpoint inhibitors. Adoptive cell therapies, such as chimeric antigen receptor (CAR) T-cell therapy, may also benefit from neutrophil modulation. By reducing the immunosuppressive influence of N2 neutrophils, the tumor microenvironment becomes more permissive to CAR-T cell infiltration and function, potentially improving outcomes in solid tumors like breast cancer^[^38^].^ Furthermore, neutrophil modulation could enhance the efficacy of cancer vaccines. Anti-tumoral N1 neutrophils can act as adjuvants, boosting the immune response to vaccine antigens and promoting a more robust cytotoxic T-cell response. This synergy could be particularly valuable in overcoming the immunosuppressive challenges posed by advanced breast cancer^[^2,38^]^.

## Recommendations

1. **Focus on Biomarker Development for Neutrophil Polarization**Research should prioritize the identification of reliable biomarkers to differentiate between N1 and N2 neutrophils in both peripheral blood and the tumor microenvironment. These biomarkers could guide patient stratification, enabling personalized therapeutic approaches that target the specific neutrophil dynamics of individual breast cancer cases.
2. **Integrate Neutrophil Modulation With Standard Therapies** Combining neutrophil-modulating agents, such as TGF-β inhibitors or CXCR2 antagonists, with standard breast cancer treatments, including immune checkpoint inhibitors and chemotherapy, could enhance therapeutic efficacy. Clinical trials should explore the timing and dosage of such combination strategies to optimize patient outcomes.
3. **Promote Research Into Epigenetic Reprogramming** The exploration of epigenetic therapies to shift neutrophil polarization from N2 to N1 should be expanded. Agents targeting histone deacetylases (HDACs) or DNA methyltransferases (DNMTs) hold promise for reprogramming neutrophil phenotypes and should be further investigated for their efficacy in breast cancer settings.
4. **Develop Advanced Drug Delivery Systems**Nanoparticle-based systems and other advanced delivery technologies should be developed to selectively target neutrophils or the tumor microenvironment. These platforms could ensure precise delivery of modulatory agents, reducing systemic toxicity and enhancing treatment specificity.
5. **Enhance Imaging Techniques for Neutrophil Tracking**Improved imaging modalities are needed to monitor neutrophil dynamics in real time within the tumor microenvironment. Such technologies could provide valuable insights into the spatiotemporal distribution of N1 and N2 neutrophils during therapy, aiding in the evaluation and adjustment of treatment protocols.
6. **Incorporate Neutrophil Analysis in Clinical Trials**Clinical trials for breast cancer therapies should include a detailed analysis of neutrophil populations as part of their design. Monitoring changes in N1/N2 ratios could serve as an indicator of therapeutic response and offer insights into resistance mechanisms.
7. **Explore Multi-Target Approaches**Therapies targeting multiple components of the tumor microenvironment, including neutrophils, macrophages, and Tregs, should be investigated. This comprehensive approach could overcome the redundancy and adaptability of immune suppression mechanisms in breast cancer.
8. **Expand Research on Combination Immunotherapies** Studies should examine how neutrophil modulation can be integrated with emerging immunotherapies, such as cancer vaccines and CAR-T cell therapies. Investigating the synergistic potential of these combinations could unlock new opportunities for tackling breast cancer.
9. **Focus on Preventive and Early Intervention Strategies**Preventive approaches, such as modulating neutrophil activity in high-risk individuals or early-stage breast cancer patients, could inhibit tumor progression and improve long-term outcomes. Research in this area could lead to the development of neutrophil-focused prophylactic treatments.
10. **Increase Awareness of Neutrophil Roles in Oncology**Educational initiatives targeting clinicians, researchers, and patients should emphasize the importance of neutrophils in cancer biology and therapy. Increased awareness could accelerate the adoption of neutrophil-based diagnostics and treatments in clinical practice.

## Conclusion

The dynamic interplay between N1 and N2 neutrophils represents a critical axis in the immune regulation of breast cancer. Once relegated to the periphery of tumor immunology, neutrophils have now taken center stage as both key modulators of the tumor microenvironment and promising therapeutic targets. The polarization of neutrophils into tumor-suppressive (N1) or tumor-promoting (N2) phenotypes is governed by a complex network of cytokines, chemokines, and metabolic cues, with factors like TGF-β, IFN-β, and CXCL family ligands playing pivotal roles. Recent research has highlighted that controlling the balance and functional plasticity of neutrophils is not only feasible but also essential for improving therapeutic responses in breast cancer – especially in aggressive and immune-evasive subtypes such as triple-negative breast cancer. The advent of targeted therapies, such as CXCR2 antagonists, TGF-β blockers, metabolic reprogramming agents, and engineered nanoparticles, has opened new clinical avenues for modulating neutrophil behavior to enhance anti-tumor immunity.

## Data Availability

Not applicable as this a narrative review.

## Research registration unique identifying number (UIN)

Not applicable as this a narrative review.

## References

[1] Testa U Castelli G Pelosi E. Breast cancer: a molecularly heterogenous disease needing subtype-specific treatments. Med Sci 2020;8:18. 10.3390/medsci8010018 PMC7151639 32210163

[2] Obeagu EI Obeagu GU. Exploring neutrophil functionality in breast cancer progression: a review. Medicine (Baltimore) 2024;103:e37654. 38552040 10.1097/MD.0000000000037654 PMC10977563

[3] Obeagu EI Obeagu GU. Breast cancer: a review of risk factors and diagnosis. Medicine (Baltimore) 2024;103:e36905. 38241592 10.1097/MD.0000000000036905 PMC10798762

[4] Jaillon S Ponzetta A Di Mitri D. Neutrophil diversity and plasticity in tumour progression and therapy. Nat Rev Cancer 2020;20:485–503. 32694624 10.1038/s41568-020-0281-y

[5] Ng LG Ostuni R Hidalgo A. Heterogeneity of neutrophils. Nat Rev Immunol 2019;19:255–65. 30816340 10.1038/s41577-019-0141-8

[6] Obeagu EI Obeagu GU. Exploring the profound link: breastfeeding’s impact on alleviating the burden of breast cancer - A review. Medicine (Baltimore) 2024;103:e37695. 38608095 10.1097/MD.0000000000037695 PMC11018178

[7] Obeagu EI Babar Q Vincent CC. Therapeutic targets in breast cancer signaling: a review. J Pharm Res Int 2021;33:82–99.

[8] Obeagu EI Obeagu GU. Beyond traditional screening: unleashing the potential of cancer antigen 27.29 for early breast cancer identification. Elite J Health Sci 2024;2:36–45.

[9] Duan J Pan L Yang M. Preoperative elevated neutrophil-to-lymphocyte ratio (NLR) and derived NLR are associated with poor prognosis in patients with breast cancer: a meta-analysis. Medicine (Baltimore) 2018;97:e13340. 30544398 10.1097/MD.0000000000013340 PMC6310509

[10] Davies AE Albeck JG. Microenvironmental signals and biochemical information processing: cooperative determinants of intratumoral plasticity and heterogeneity. Front Cell Dev Biol 2018;6:44. 29732370 10.3389/fcell.2018.00044 PMC5921997

[11] Ondeck MG Kumar A Placone JK. Dynamically stiffened matrix promotes malignant transformation of mammary epithelial cells via collective mechanical signaling. Proc National Acad Sci 2019;116:3502–07. 10.1073/pnas.1814204116 PMC6397509 30755531

[12] Nicolini A Ferrari P Carpi A. Immune checkpoint inhibitors and other immune therapies in breast cancer: a new paradigm for prolonged adjuvant immunotherapy. Biomedicines 2022;10:2511. 36289773 10.3390/biomedicines10102511 PMC9599105

[13] Hristova-Panusheva K Xenodochidis C Georgieva M. Nanoparticle-mediated drug delivery systems for precision targeting in oncology. Pharmaceuticals 2024;17:677. 38931344 10.3390/ph17060677 PMC11206252

[14] Su Y Gao J Kaur P. Neutrophils and macrophages as targets for development of nanotherapeutics in inflammatory diseases. Pharmaceutics 2020;12:1222. 33348630 10.3390/pharmaceutics12121222 PMC7766591

[15] Zhang S Meng Y Zhou L. Targeting epigenetic regulators for inflammation: mechanisms and intervention therapy. MedComm 2022;3:e173. 36176733 10.1002/mco2.173 PMC9477794

[16] Wu G Pan B Shi H. Neutrophils’ dual role in cancer: from tumor progression to immunotherapeutic potential. Int Immunopharmacol 2024;140:112788. 39083923 10.1016/j.intimp.2024.112788

[17] Awasthi D Sarode A. Neutrophils at the crossroads: unraveling the multifaceted role in the tumor microenvironment. Int J Mol Sci 2024;25:2929. 38474175 10.3390/ijms25052929 PMC10932322

[18] Ohms M Möller S Laskay T. An attempt to polarize human neutrophils toward N1 and N2 phenotypes in vitro. Front Immunol 2020;11:532. 32411122 10.3389/fimmu.2020.00532 PMC7198726

[19] Ma Y Yabluchanskiy A Iyer RP. Temporal neutrophil polarization following myocardial infarction. Cardiovasc Res 2016;110:51–61. 26825554 10.1093/cvr/cvw024 PMC4798046

[20] Fridlender ZG Sun J Kim S. Polarization of tumor-associated neutrophil phenotype by TGF-β:“N1” versus “N2” TAN. Cancer Cell 2009;16:183–94. 19732719 10.1016/j.ccr.2009.06.017 PMC2754404

[21] Lovászi M Németh ZH Pacher P. A2A adenosine receptor activation prevents neutrophil aging and promotes polarization from N1 towards N2 phenotype. Purinergic Signal 2022;18:345–58. 35838900 10.1007/s11302-022-09884-0 PMC9391554

[22] Sansores-España LD Melgar-Rodríguez S Vernal R. Neutrophil N1 and N2 subsets and their possible association with periodontitis: a scoping review. Int J Mol Sci 2022;23:12068. 36292925 10.3390/ijms232012068 PMC9603394

[23] McFarlane AJ Fercoq F Coffelt SB. Neutrophil dynamics in the tumor microenvironment. J Clin Invest 2021;131:e143759. 33720040 10.1172/JCI143759 PMC7954585

[24] Liang B Yuan Y Jiang Q. How neutrophils shape the immune response of triple-negative breast cancer: novel therapeutic strategies targeting neutrophil extracellular traps. Biomed Pharmacother 2024;178:117211. 39068851 10.1016/j.biopha.2024.117211

[25] Gazinska P Milton C Iacovacci J. Dynamic changes in the NK-, neutrophil-, and B-cell immunophenotypes relevant in high metastatic risk post neoadjuvant chemotherapy–resistant early breast cancers. Clin Cancer Res 2022;28:4494–508. 36161312 10.1158/1078-0432.CCR-22-0543 PMC9561554

[26] SenGupta S Hein LE Xu Y. Triple-negative breast cancer cells recruit neutrophils by secreting TGF-β and CXCR2 ligands. Front Immunol 2021;12:659996. 33912188 10.3389/fimmu.2021.659996 PMC8071875

[27] Ho T Clermont G Parker RS. A model of neutrophil dynamics in response to inflammatory and cancer chemotherapy challenges. Comput Chem Eng 2013;51:187–96.

[28] Chen Q Yin H Liu S. Prognostic value of tumor-associated N1/N2 neutrophil plasticity in patients following radical resection of pancreas ductal adenocarcinoma. J Immunother Cancer 2022;10:e005798. 36600557 10.1136/jitc-2022-005798 PMC9730407

[29] Tyagi A Sharma S Wu K. Nicotine promotes breast cancer metastasis by stimulating N2 neutrophils and generating pre-metastatic niche in lung. Nat Commun 2021;12:474. 33473115 10.1038/s41467-020-20733-9 PMC7817836

[30] Grecian R Whyte MK Walmsley SR. The role of neutrophils in cancer. Br Med Bull 2018;128:5–14. 30137312 10.1093/bmb/ldy029 PMC6289220

[31] de Los Reyes AA Kim Y. Optimal regulation of tumour-associated neutrophils in cancer progression. R Soc Open Sci 2022;9:210705. 35127110 10.1098/rsos.210705 PMC8808100

[32] Saraiva DP Correia BF Salvador R. Circulating low density neutrophils of breast cancer patients are associated with their worse prognosis due to the impairment of T cell responses. Oncotarget 2021;12:2388. 34853660 10.18632/oncotarget.28135 PMC8629401

[33] Hajizadeh F Maleki LA Alexander M. Tumor-associated neutrophils as new players in immunosuppressive process of the tumor microenvironment in breast cancer. Life Sci 2021;264:118699. 33137368 10.1016/j.lfs.2020.118699

[34] Coffelt SB Wellenstein MD de Visser KE. Neutrophils in cancer: neutral no more. Nat Rev Cancer 2016;16:431–46. 27282249 10.1038/nrc.2016.52

[35] Luyang H Zeng F Lei Y. Bidirectional role of neutrophils in tumor development. Mol Cancer 2025;24:22. 39819428 10.1186/s12943-025-02228-7 PMC11737241

[36] Obeagu EI Rizvi SA. Inflammatory signaling pathways in neutrophils: implications for breast cancer therapy. Ann Med Surg 2025;87:10–97. 10.1097/MS9.0000000000003251 PMC12140771 40486614

[37] Yang S Jia J Wang F. Targeting neutrophils: mechanism and advances in cancer therapy. Clin Transl Med 2024;14:e1599. 38450975 10.1002/ctm2.1599 PMC10918741

[38] Zhang S Sun L Zuo J. Tumor associated neutrophils governs tumor progression through an IL-10/STAT3/PD-L1 feedback signaling loop in lung cancer. Transl Oncol 2024;40:101866. 38128466 10.1016/j.tranon.2023.101866 PMC10753083

[39] Yang C Li L Ye Z. Mechanisms underlying neutrophils adhesion to triple-negative breast cancer cells via CD11b-ICAM1 in promoting breast cancer progression. Cell Commun Signal 2024;22:340. 38907234 10.1186/s12964-024-01716-5 PMC11191284

[40] Yang C Wang Z Li L. Aged neutrophils form mitochondria-dependent vital NETs to promote breast cancer lung metastasis. J Immunother Cancer 2021;9:e002875. 34716206 10.1136/jitc-2021-002875 PMC8559246

[41] Ko K Woo SW Chae YC. Potential involvement of neutrophils on exercise effects in breast cancer malignancy. Phys Act Nutr 2023;27:41. 10.20463/pan.2023.0036 PMC10844724 38297475

[42] Lau D Lechermann LM Gallagher FA. Clinical translation of neutrophil imaging and its role in cancer. Mol Imaging Biol 2022;24:221–34. 34637051 10.1007/s11307-021-01649-2 PMC8983506

[43] Chung AW Anand K Anselme AC. A phase 1/2 clinical trial of the nitric oxide synthase inhibitor L-NMMA and taxane for treating chemoresistant triple-negative breast cancer. Sci Transl Med 2021;13:eabj5070. 34910551 10.1126/scitranslmed.abj5070

[44] Zheng C Xu X Wu M. Neutrophils in triple-negative breast cancer: an underestimated player with increasingly recognized importance. Breast Cancer Res 2023;25:88. 37496019 10.1186/s13058-023-01676-7 PMC10373263

[45] Rui XU Zehao WA Jiong WU. Advances in the role of tumor-associated neutrophils in the development of breast cancer. China Oncol 2024;34:881–89.

[46] Ren K He J Qiu Y. A neutrophil-mediated carrier regulates tumor stemness by inhibiting autophagy to prevent postoperative triple-negative breast cancer recurrence and metastasis. Acta Biomater 2022;145:185–99. 35447368 10.1016/j.actbio.2022.04.017

[47] Akinsipe T Mohamedelhassan R Akinpelu A. Cellular interactions in tumor microenvironment during breast cancer progression: new frontiers and implications for novel therapeutics. Front Immunol 2024;15:1302587. 38533507 10.3389/fimmu.2024.1302587 PMC10963559

[48] Huang YC Chang CY Wu YY. Single-cell transcriptomic profiles of lung pre-metastatic niche reveal neutrophil and lymphatic endothelial cell roles in breast cancer. Cancers (Basel) 2022;15:176. 36612175 10.3390/cancers15010176 PMC9818165

[49] Al Qutami F AlHalabi W Vijayakumar A. Characterizing the inflammatory profile of neutrophil-rich triple-negative breast cancer. Cancers (Basel) 2024;16:747. 38398138 10.3390/cancers16040747 PMC10886617

[50] Zhao J Xie X. Prediction of prognosis and immunotherapy response in breast cancer based on neutrophil extracellular traps-related classification. Front Mol Biosci 2023;10:1165776. 37304069 10.3389/fmolb.2023.1165776 PMC10250592

[51] Munkácsy G Santarpia L Győrffy B. Therapeutic potential of tumor metabolic reprogramming in triple-negative breast cancer. Int J Mol Sci 2023;24:6945. 37108109 10.3390/ijms24086945 PMC10138520

[52] Ralph SJ Reynolds MJ. Intratumoral pro-oxidants promote cancer immunotherapy by recruiting and reprogramming neutrophils to eliminate tumors. Cancer Immunol Immunother 2023;72:527–42. 36066649 10.1007/s00262-022-03248-8 PMC9446783

[53] Kaisar-Iluz N Arpinati L Shaul ME. The bilateral interplay between cancer immunotherapies and neutrophils’ phenotypes and Sub-populations. Cells 2022;11:783. 35269405 10.3390/cells11050783 PMC8909700

[54] Shaul ME Levy L Sun J. Tumor-associated neutrophils display a distinct N1 profile following TGFβ modulation: a transcriptomics analysis of pro-vs. antitumor TANs. Oncoimmunology 2016;5:e1232221. 27999744 10.1080/2162402X.2016.1232221 PMC5139653

[55] Oberg HH Wesch D Kalyan S. Regulatory interactions between neutrophils, tumor cells and T cells. Front Immunol 2019;10:1690. 31379875 10.3389/fimmu.2019.01690 PMC6657370

[56] Barreno L Sevane N Valdivia G. Transcriptomics of canine inflammatory mammary cancer treated with empty cowpea mosaic virus implicates neutrophils in anti-tumor immunity. Int J Mol Sci 2023;24:14034. 37762335 10.3390/ijms241814034 PMC10531449

